# Two-dimensional mass spectrometry: new perspectives for tandem mass spectrometry

**DOI:** 10.1007/s00249-019-01348-5

**Published:** 2019-03-13

**Authors:** Maria A. van Agthoven, Yuko P. Y. Lam, Peter B. O’Connor, Christian Rolando, Marc-André Delsuc

**Affiliations:** 1 Department of Chemistry, University of Warwick, Gibbet Hill Road, Coventry, CV47AL UK; 2 MSAP USR 3290, Université Lille, Sciences et Technologies, 59655 Villeneuve d’Ascq Cedex, France; 3 Institut de Génétique, Biologie Moléculaire et Cellulaire, INSERM, U596, CNRS, UMR7104, Université de Strasbourg, 1 rue Laurent Fries, 67404 Illkirch-Graffenstaden, France; 4 CASC4DE, 20 avenue du Neuhof, 67100 Strasbourg, France

**Keywords:** Mass spectrometry, Two dimensional, Fourier transform, Fourier transform ion cyclotron resonance mass spectrometry, Tandem mass spectrometry

## Abstract

Fourier transform ion cyclotron resonance mass analysers (FT-ICR MS) can offer the highest resolutions and mass accuracies in mass spectrometry. Mass spectra acquired in an FT-ICR MS can yield accurate elemental compositions of all compounds in a complex sample. Fragmentation caused by ion–neutral, ion–electron, or ion–photon interactions leads to more detailed structural information on compounds. The most often used method to correlate compounds and their fragment ions is to isolate the precursor ions from the sample before fragmentation. Two-dimensional mass spectrometry (2D MS) offers a method to correlate precursor and fragment ions without requiring precursor isolation. 2D MS therefore enables easy access to the fragmentation patterns of all compounds from complex samples. In this article, the principles of FT-ICR MS are reviewed and the 2D MS experiment is explained. Data processing for 2D MS is detailed, and the interpretation of 2D mass spectra is described.

## Introduction

In mass spectrometry, samples are ionized, separated according to mass-to-charge (*m*/*z*) ratio by modifying their trajectories and their velocities in an electric or magnetic field, and detected (de Hoffmann and Stroobant 2007). The measurement of their *m*/*z* ratio gives information about the elemental composition of the compounds in the samples: the more accurate the *m*/*z* ratio measurement, the more certainty there is about the elemental composition of the compounds. To quantify the confidence of the data interpretation, the resolution and the resolving power of individual peaks or mass spectra are measured. In the IUPAC definition, the resolving power of a peak in a mass spectrum refers to the smallest mass difference between two peaks that can be differentiated. The resolution measures the ratio between the mass measurement and the resolving power. The accuracy of a mass assignment for a peak refers to the difference between the experimental and theoretical *m*/*z* ratios divided by the theoretical *m*/*z* ratio (Marshall et al. 1998).

In addition to measuring the *m*/*z* ratio of compounds, mass spectrometers can also be used to fragment them to elucidate their chemical structure (tandem mass spectrometry) (Biemann 2015). Current fragmentation methods focus mainly on interactions with neutral particles, photons, and electrons. The ions from the sample are called precursors and the ions that are products of the fragmentation are called fragments. One important problem in tandem mass spectrometry of complex samples is how to correlate precursors and fragments to elucidate precursor structures accurately. The most common way to correlate precursors and fragments is to isolate a single *m*/*z* ratio before fragmenting it (MS/MS) (Biemann 2002). MS/MS can be coupled with separation techniques to reduce the instantaneous complexity of the sample and to reduce ionization competition and Coulombic repulsion between ions (Peng et al. 2003; Ruger et al. 2017; Wootton et al. 2017). Because a choice is made for each MS/MS spectrum on which *m*/*z* ratio is isolated and fragmented (a decision that is usually made algorithmically by the computer running the instrument: the acquisition switches between MS and MS/MS. In MS acquisition, the peaks with the highest intensity are identified and chosen to be isolated and fragmented. A mass exclusion list is generated and updated at every MS scan, so that a given ion species is not isolated and fragmented twice during the LC–MS/MS run), MS/MS and MS/MS coupled with separation is called a data-dependent acquisition (or DDA) technique (Decaestecker et al. 2000). DDA is a very established technique that is well adapted to targeted analysis, in which the compounds of interest are determined before the analysis, for example in the case of pesticide analysis determined by regulations (Chapman et al. 2014).

However, for complex samples with multiple co-eluting species requiring analysis, data-independent acquisition (DIA) methods have been developed. One such method is precursor acquisition independent from ion count (PAcIFIC): ions go through a quadrupole in which a predetermined mass range is isolated, the isolated ions are then fragmented and analysed, and the mass range that is isolated is regularly incremented after each scan (Chapman et al. 2014; Panchaud et al. 2009). A similar technique has been commercialized as sequential windowed acquisition of all theoretical fragment ion mass spectra (SWATH-MS) by Sciex (Gillet et al. 2012). Another DIA technique, called MS^E^, developed and commercialized by Waters, takes advantage of the fast duty cycle of time-of-flight analysers (1 ms) compared to the length of a chromatographic peak (10 s) by acquiring mass spectra with low and high fragmentation energy to correlate precursor and fragments (Cramer et al. 2016; Plumb et al. 2006).

A major issue of PAcIFIC/SWATH-MS is its reliance on ion isolation, which leads to experiment times and sample consumption that increase when the quadrupole isolation window (PAcIFIC) or the step size (SWATH) is decreased. Furthermore, because the width of the isolation windows is typically over 2 Da, separation between overlapping isotopic distributions with these methods is very difficult. In this paper, the progress of an alternative DIA method, two-dimensional mass spectrometry (2D MS), is reviewed (van Agthoven et al. 2013). First, the general theory of the mass analyser on which 2D MS is developed, Fourier transform ion cyclotron resonance mass spectrometry (FT-ICR MS), is presented. Second, the principles of the 2D MS experiment and data processing are explained. Third, the interpretation of a 2D mass spectrum and the applications of 2D MS are described.

## Fourier transform ion cyclotron resonance mass spectrometry and tandem mass spectrometry

**Fig. 1:**
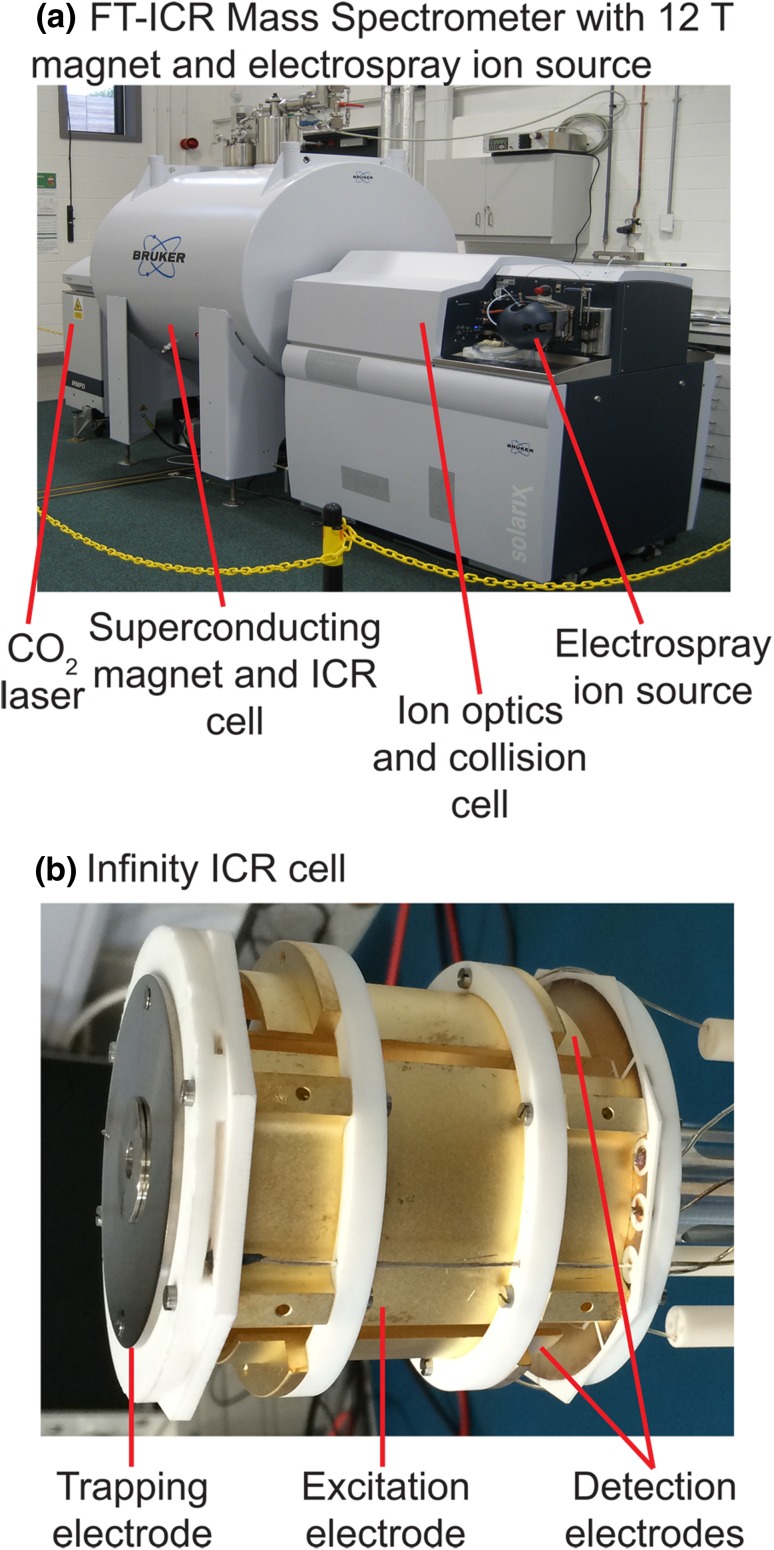
**a** Photograph of a 12 T FT-ICR mass spectrometer with an electrospray ion source. Samples are ionized in the electrospray source and travel to the ICR cell in the centre of the superconducting magnet. The CO_2_ laser is used for fragmentation in the ICR cell. **b** Photograph of an Infinity ICR cell. The trapping electrodes are used to trap the ions axially. The excitation electrodes excite the ions to high radii and the detection electrodes detect mirror current generated by their motion

Figure 1a shows a photograph of an FT-ICR mass spectrometer. The ion source is external to the mass analyser and ions are transferred from the ion source to the mass analyser through ion optics. The mass analyser is an ion cyclotron resonance (ICR) cell situated in the centre of a superconducting magnet. A photograph of an ICR cell is shown in Fig. 1b.

FT-ICR mass spectrometry uses the cyclotron motion of ions inside a magnetic field. A homogeneous magnetic field induces a circular motion in ions with a frequency that can be expressed as:

1 $$\omega_{\text{c}} = \frac{qB}{m},$$

in which *ω*_c_ is the cyclotron frequency, *B* the magnetic field, *q* the charge of the ion and *m* its mass. FT-ICR MS works by measuring the frequencies of ion motions in a homogeneous magnetic field and converting them into *m*/*z* ratios (Amster 1996; Marshall et al. 1998). While Eq. 1 gives a good approximation (to 1 part in 10^4^ typically) of the frequency, the measured frequency will also be slightly shifted by the trapping electric fields, the pressure, and the Coulombic repulsion electric fields of the other ions that are simultaneously trapped. The resulting ‘corrected’ frequency is called the reduced cyclotron frequency and is fully considered in the true calibration equations (Zhang et al. 2005).

**Fig. 2:**
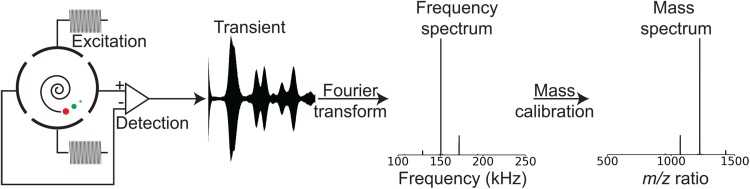
Acquisition and processing of a mass spectrum with an FT-ICR mass spectrometer. The mirror current is measured between the detection electrodes, amplified and converted into a voltage, and acquired. A FT yields the frequency spectrum, and a calibration by the frequency-to-mass conversion yields the mass spectrum

Ions are trapped inside an ICR cell made up of trapping electrodes, excitation electrodes, and detection electrodes (Vartanian et al. 1995), as shown in Fig. 1b. Figure 2 shows the process of acquiring and processing a mass spectrum inside an FT-ICR MS. In the simplest configuration, there are two electrodes for each function. Ions are trapped radially by the magnetic field and axially by the trapping electrodes. Ion packets in proximity to a detection plate interact with the electrons in that plate. The current between the two detection plates, called the mirror current, is amplified, converted into a voltage, and measured at regular time intervals. This measurement is called the transient, and its Fourier transform yields the cyclotron frequencies of the ions. Frequencies can then be converted into *m*/*z* ratios.

Because the natural radius of ions over the mass range of analytical interest (< 1 mm) is very small compared to the radius of the ICR cell (3 cm), and because the ions’ initial phases are random (i.e. incoherent), causing destructive interference of any tiny electrical signals they do generate, the mirror current is vanishingly small when ions are at rest in the ICR cell. Therefore, ions are resonantly excited with a radiofrequency voltage applied to the excitation plates, as shown in Fig. 2. Typically, the mass range of interest is between *m*/*z* 100 and *m*/*z* 3000. The corresponding frequency range is between 2 MHz and 35 kHz (which is ~ 100 Da per charge and ~ 3000 Da per charge respectively at 7 T). The radiofrequency voltage is therefore a broadband excitation (Comisarow and Marshall 1974). The cyclotron radius of the ions at the end of the excitation is proportional to the amplitude and the duration of the radiofrequency voltage and does not depend on *m*/*z* ratio (Gorshkov and Nikolaev 1993).

The two methods to generate excitation voltages are frequency sweep excitation and stored waveform inverse Fourier transform (SWIFT) (Comisarow and Marshall 1974; Wang et al. 1986). Frequency sweep excitation consists in applying a sinusoidal voltage with a variable frequency that sweeps through the frequency range. In SWIFT, the inverse Fourier transform of the desired frequency envelope is calculated to generate the time domain waveform that is to be applied to the excitation plates. A phase function can be calculated to spread out the signal in the time domain and reduce the maximum voltage on the excitation plates (Guan and McIver 1990).

After excitation, ions form coherent packets which rotate at their reduced cyclotron frequency (Nikolaev et al. 2007), which corresponds to the frequency of the cyclotron motion of the ions plus the frequency of the motion caused by the trapping potential. Ion packet coherence enables mirror current detection between the detection plates, because at each moment during detection all ions of a given *m*/*z* ratio are closer to one detection plate than the other. The mirror current is measured at regular intervals (shorter than twice the period of the orbit of the fastest ion), converted into a voltage and amplified (Comisarow 1978; Jerri 1977). The resulting measurement is called the transient (see Fig. 2).

Ion packet coherence can be destroyed by Coulombic repulsion, which limits the number of ions in the ICR cell, or by collisions with residual background gas in the ICR cell (Aizikov et al. 2009; Boldin and Nikolaev 2009). To maintain ion packet coherence as long as possible in the ICR cell, the pressure in the ICR cell needs to be between 10^−10^ and 10^−9^ Torr at all times. In these vacuum conditions, ion packet coherence can last longer than 1 s and up to several minutes with careful experimental tuning.

Fourier transformation of the transient leads to a frequency spectrum showing peaks at the frequencies of the ions (see Fig. 2). Long transients of over 1 s lead to frequency measurement precisions of lower than 1 Hz (Nikolaev et al. 2011). For an ion at *m*/*z* 400 rotating in a magnetic field of 12 T, the cyclotron frequency is 460 kHz. This leads to resolving power at *m*/z 400 that easily exceeds 230,000 (in 1 s) and mass accuracies better than 1 ppm (Nikolaev et al. 2012). Provided that the ion number is kept low enough that Coulombic repulsion amongst the ions is minimal, the main factors determining the resolving power in an FT-ICR mass spectrum are the magnetic field, the transient duration (itself limited by the magnetic field), the pressure inside the ICR cell, and the acquisition time (Amster 1996; Marshall et al. 1998).

The mass spectrum is obtained by converting the reduced cyclotron frequencies into *m*/*z* ratios. The high resolving power of the frequency spectrum leads to high resolving powers in the mass spectra as well. In a mass spectrum, because the *m*/*z* ratio is in first approximation inversely proportional to the frequency, the resolving power decreases with increase in *m*/*z* ratios. FT-ICR MS is considered to be the mass analyser with the highest resolution and mass accuracy of all (Marshall and Hendrickson 2008; Valeja et al. 2011; Wei et al. 2016). Nevertheless, in the Orbitrap mass analyser, the resolving power decreases with the square root of the *m*/*z* ratio, which means that at high *m*/*z* ratios, Orbitraps may have higher resolving powers than FT-ICR mass analysers (Denisov et al. 2012).

A first approximation of the frequency-to-mass conversion can be obtained using Eq. 1. The use of a trapping voltage between the two trapping electrodes changes the frequency-to-mass conversion because the measured frequency is the reduced cyclotron frequency instead of the cyclotron frequency. Linear or quadratic functions are therefore most often used to convert frequencies into *m*/*z* ratios (Francl et al. 1983; Ledford et al. 1984; Shi et al. 2000). In most FT-ICR mass spectrometers, the instrument is calibrated daily using a standard compound mixture to correct the effect of the magnetic field drift (typically of the order of a ppb/day). Internal calibration of mass spectra uses the measured frequency of the peaks at their centroid and the theoretical *m*/*z* ratio of the compounds they are assigned to. A typical internal calibration uses a quadratic frequency-to-mass conversion:

2 $$m/z = \frac{A}{{\omega^{2} }} + \frac{B}{\omega } + C,$$

in which *m/z* is the theoretical *m/z* ratio, *ω* the measured frequency of the peak at its centroid, and *A*, *B*, and *C* are constants that are calculated using a quadratic regression (Ledford et al. 1984). If the assignments of the peaks are correct, then internal calibration reduces the mass accuracies of the peak assignments.

For tandem mass spectrometry, there are multiple isolation methods in an FT-ICR mass spectrometer. Because of the pressure constraints, the ion source is external to the ICR cell and ions are transported through ion optics to the ICR cell. The ion optics often consist of electrodes that form a multipolar electric field, whose voltages can be manipulated to enable trajectory stability for ions within a mass window of 1 Da or less, therefore enabling ion isolation in the front end of the FT-ICR mass spectrometer (Gerlich 1992; Jebanathirajah et al. 2005; O’Connor et al. 2006). Ion isolation can also be achieved in the ICR cell by exciting all ions except the ion of interest radially up to the electrodes with a resonant excitation. Both a frequency sweep that skips the frequencies of the ions of interest (CHEF) and a SWIFT excitation with a notched frequency envelope can be used for this purpose (de Koning et al. 1997; McDonald et al. 2003; O’Connor et al. 1996; O’Connor and McLafferty 1995).

The most popular fragmentation method is collision-induced dissociation (CID, also called collisionally activated dissociation, or CAD), which consists in non-elastic collisions between ions and a neutral gas. During each collision, part of the kinetic energy is converted into internal energy, which leads to the fragmentation of the bonds with the lowest dissociation energy in the ion (Sleno and Volmer 2004). The main parameters used to control the degree of dissociation are the nature of the neutral gas (the most often used gas is argon), its pressure in the collision cell, and the kinetic energy of the ions in the collision cell.

Infrared multiphoton dissociation (IRMPD) relies on the absorption of infrared photons by ions (Little et al. 1994). The IR photons are often generated by a CO_2_ laser with a 10.6 µm wavelength (Garnier et al. 2009). The absorption of IR photons increases the internal energy of the ions, and the bonds with the lowest energy rearrangements are dissociated (Ramaswamy et al. 1981). Another photon-based fragmentation method is ultraviolet photodissociation (UVPD), in which a UV photon generated by an NdYAG laser (266 nm or 213 nm) or an excimer laser (193 nm) is absorbed by ions (Agarwal et al. 2011; Madsen et al. 2010; Racaud et al. 2009). In both IRMPD and UVPD, the fragmentation efficiency depends on the presence of a chromophore in the ion.

The first electron-based fragmentation method is electron capture dissociation (ECD), in which multiply charged positive ions capture one or more electrons generated by an electron gun, which destabilizes the structure of the ion and leads to fragmentation (Kruger et al. 1999; Zubarev et al. 1998). However, because of mass differences between ions and electrons and the voltages typically applied to ion trap electrodes, electrons and ions can only be trapped simultaneously in a high magnetic field, which means that ECD is most practicable in FT-ICR mass spectrometers. Although ECD can only be used with positive ions with multiple charges, other electron-based fragmentation methods have been developed to achieve fragmentation of singly charged positive ions (electron-induced dissociation, EID, or electron impact excitation of ions from organics, or EIEIO) (Cody and Freiser 1979; Haselmann et al. 2002; Mosely et al. 2011; Wei et al. 2013; Wills and O’Connor 2014). To fragment negative ions, electron-detachment dissociation (EDD) and negative ion ECD (niECD) have been developed (Budnik et al. 2001; Kaczorowska and Cooper 2008; Song and Hakansson 2012).

The fragmentation methods listed in the previous paragraph can be performed inside the ICR cell. Laser-based dissociation methods like IRMPD and UVPD are performed at the centre of the ICR cell. Electron-based dissociation methods have a fragmentation zone that depends on the shape of the electron gun. For example, in the case of a hollow cathode, the shape of the fragmentation zone has a maximum at a non-zero radius, but has significant fragmentation efficiency at the centre of the ICR cell (Tsybin et al. 2003). Because of the pressure constraints in the ICR cell, fragmentation methods that require gas, like CAD or electron-transfer dissociation (ETD), are mostly performed in a collision cell in the front end of the mass spectrometer (Syka et al. 2004).

Being able to use various fragmentation methods to perform tandem mass spectrometry can yield complementary structural information. For example, in the case of peptides and proteins, IRMPD preferentially fragments post-translational modifications such as glycosylations and phosphorylations, whereas ECD preferentially fragments the amino acid backbone (Hakansson et al. 2001). Fragmenting a peptide or a protein with both ECD and IRMPD can therefore increase the amount of structural information available on them.

Because of its transient duration, the duty cycle of an FT-ICR mass spectrometer (i.e. the time necessary to acquire a single mass spectrum) is of the order of 1 s. FT-ICR mass spectrometers can therefore be coupled with chromatographic methods. However, DIA methods such as PAcIFIC, SWATH, or MS^E^, depend on mass analysers with duty cycles of the order of 1 ms. Coupling these DIA methods with an FT-ICR mass spectrometer would lead to very long experiments, high sample consumption, and very large datasets.

### Principle of two-dimensional mass spectrometry

In 1984, Marshall et al. demonstrated the reversibility of resonant excitation inside the ICR cell. In a first experiment, ions were excited with a single frequency resonant excitation with a constant amplitude and increasing duration (Marshall et al. 1984). Since the radius of the ions after excitation increased, the intensity of the peak in the mass spectrum increased until the ion radius reached the radius of the ICR cell. In a second experiment, the phase of the resonant excitation was switched by 180° after 1 ms, by inversing the sign of the applied voltage. A decrease of the peak was observed until an excitation duration of 2 ms, when the peak intensity was zero. In the first experiment, the ion packet remained closest to the excitation plate with the attractive potential, which caused the radius to increase. In the second experiment, after the phase switch, the ions were closest to the excitation plate with the repelling voltage, which caused the radius to decrease. After 2 ms, the ions were back at the centre of the ICR cell, and therefore undetectable. This experiment proved that ion excitation was reversible in the ICR cell.

**Fig. 3:**
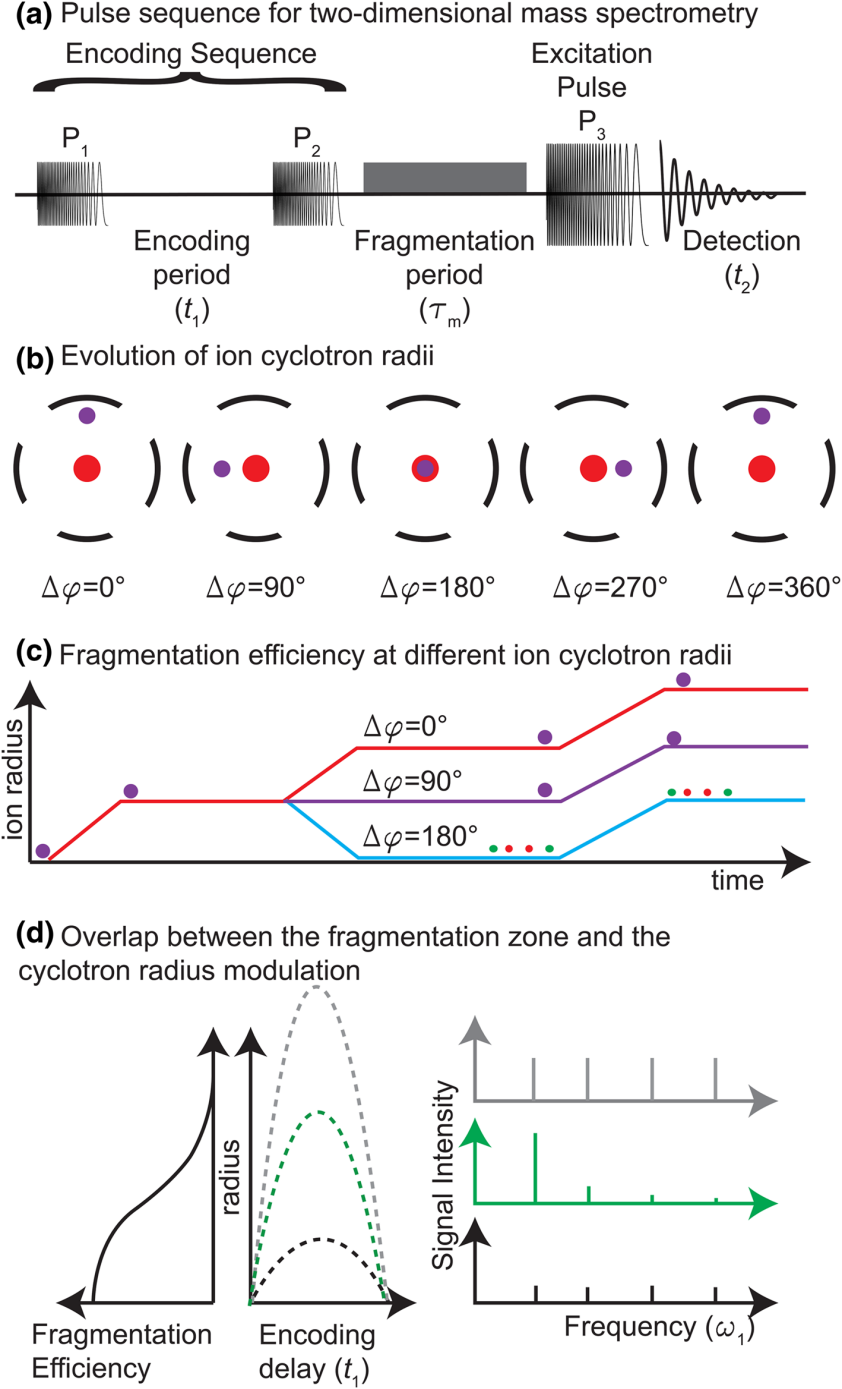
**a** Pulse sequence for a 2D MS experiment. The encoding sequence modulates the radius of the ions according to their cyclotron frequency and the delay *t*_1_. The radius-dependent fragmentation then modulates the abundance of the precursors and all ions are excited and detected. **b** Evolution of ion cyclotron radii during a 2D MS experiment. According to the product of their cyclotron frequency and the delay *t*_1_, the ion packets have different radii at the end of the encoding sequence. **c** Fragmentation efficiency at different cyclotron radii. The cyclotron radii of the ions after the encoding sequence determines the fragmentation efficiency. **d** Overlap between the fragmentation zone and the radius modulation. There is an optimal overlap between the fragmentation zone and the amplitude of the cyclotron radius modulation where multiple *ω*_1_ harmonics are minimized

In 1987, Pfändler et al. proposed a pulse sequence for two-dimensional mass spectrometry based on Marshall’s conclusions (Pfändler et al. 1987). This pulse sequence is shown in Fig. 3a. The first pulse P_1_ excites ions from the centre of the ICR cell to a radius *r*_1_. During the delay *t*_1_, the phase of the cyclotron motion of the ions increases by *ω*_ICR_ × *t*_1_, in which *ω*_ICR_ is the cyclotron motion of the ions. After *t*_1_, a pulse P_2_ which is identical to P_1_ is applied. The frequency range of P_1_ and P_2_ needs to cover the cyclotron frequency range of all the precursor ions of interest in the sample.

If *φ*_ion_ is the phase of the ion trajectory in the ICR cell in polar coordinates around the axis at the centre of the ICR cell and *φ*_RF_ is the phase of the RF voltage, then the difference *Δφ* between the two at the end of P_2_ can be expressed as:

3 $$\Delta \varphi = \varphi_{\text{ion}} - \varphi_{\text{RF}} = \left( {\omega_{\text{ICR}} - \omega_{\text{N}}^{\text{RF}} } \right)t_{1} + \Delta \psi ,$$

in which *ω*_N_^RF^ is the last frequency in the P_1_ pulse and *Δψ* is a constant that has been shown by Pfändler et al. to only depend on the characteristics of P_1_ and P_2_ (Pfändler et al. 1988). The term − *ω*_N_^RF^ is needed when a continuous phase pulse generator is used (this is the case for pulse generators in Bruker FT-ICR mass spectrometers, which are the most popular commercial instruments). In this case the generator keeps its central clock oscillating at the lowest generated frequency and generates the P_2_ pulse with the accumulated phase of this frequency over the *t*_1_ period. A non-continuous phase generator may cause other difficulties which may hamper altogether the possibility of recording the modulation during *t*_1_. The radius at the end of P_2_ can be expressed as:

4 $$r\left( {t_{1} } \right) = r_{1} \sqrt {2\left( {1 + \cos \omega_{A} \left( {t_{1} - T} \right)} \right)} ,$$

in which $\omega_{\text{A}} = \omega_{\text{ICR}} - \omega_{\text{N}}^{\text{RF}}$, *r*_1_ is the radius of the ions at the end of P_1_, and *T* is the duration of P_1_ (Guan and Jones 1989).

Figure 3b shows the cyclotron radii of the ions at the end of P_2_ for different values of *Δφ* (cf. Eq. 3). If *Δφ *= 0°, then ions are attracted by the excitation electrode they are closest to during resonant excitation in P_2_. The ion packet is therefore excited to double their radius from what it was at the end of P_1_. If *Δφ *= 180°, then ions are repelled by the excitation electrode they are closest to during resonant excitation in P_2_. The effect of P_2_ therefore is that the ion packet is de-excited and goes back to the centre of the ICR cell. If *Δφ *= 90° or *Δφ *= 270°, then the ion packet is at equal distance from either excitation electrode during resonant excitation in P_2_, and P_2_ has no effect on the ion packet radius. When *Δφ *= 360°, the situation is identical to the *Δφ *= 0° situation. After radius modulation of the precursor ions, a fragmentation mode with radius-dependent fragmentation efficiency is applied for a duration of *τ*_m_. Figure 3c shows how the ion packet radius at the end of P_2_ affects fragmentation efficiency. The diagram in Fig. 3c is reminiscent of coherence transfer pathway diagrams used in nuclear magnetic resonance spectroscopy (NMR), which display the coherence order explored by the spin system while pulses are applied to the sample (Bodenhausen et al. 1984). In NMR, the quantum state can only take discrete values, which can also be negative. However, both diagrams share many properties: the initial state is always at zero; only pulses may modify the level value; this value describes the system state; the final state, where acquisition of the signal occurs, has to be on a special value (− 1 in NMR vs. a narrow range of radii in 2D FT-ICR).

For fragmentation methods like ion–molecule reactions or CAD, the fragmentation efficiency increases with the internal energy of the precursor ions after collision, i.e. with their kinetic energy when collisions happen. In the ICR cell, after radius modulation in a 2D FT-ICR MS experiment, the kinetic energy of the precursor ions, and therefore their fragmentation efficiency, is proportional to *r*^2^(*t*_1_) (Bensimon et al. 1989).

In IRMPD, the laser is aligned with the axis at the centre of the ICR cell. Ions absorb multiple photons which increase their kinetic energy until fragmentation happens. Fragmentation efficiency is therefore proportional to photon density and decreases when precursor ion radii increase. In IRMPD, occurrences of secondary fragmentation (i.e. fragment ions that absorb photons and then fragment) need to be considered (van Agthoven et al. 2011b). In ECD, the fragmentation efficiency depends on the electron density in the ICR cell, which depends on the shape of the electron emitter. Broadly speaking, the electron density is largest close to the centre of the ICR cell, and fragmentation efficiency decreases when the precursor ion radius increases (van Agthoven et al. 2012).

Because the presence of gas in the ICR cell destroys ion packet coherence, CAD is not a fragmentation method that is compatible with 2D MS. IRMPD and ECD do not require any gas, and so they are the most used. When *Δφ* is at a value (e.g. 0° or 90°, or red and purple lines in Fig. 3c, for IRMPD) where the ions are outside of the fragmentation zone (red zone in Fig. 3b), then no fragmentation occurs during *τ*_m_. When *Δφ* is at a value (e.g. close to 180°, or blue line in Fig. 3c, for IRMPD) where the ions are within the fragmentation zone, then fragmentation occurs during *τ*_m_. After *τ*_m_, all ions (unfragmented precursors and fragments) are excited and detected. A transient is recorded for each value of *t*_1_. The precursor ions are modulated in and out of the fragmentation zone at different frequencies, and the fragment ion abundance therefore carries the modulation frequency of the precursor ions.

In the 2D MS pulse sequence, the ion radius modulation *r*_1_ has to be optimized according to the size of the fragmentation zone. Figure 3d shows how the overlap between the radius modulation and the fragmentation zone affects the 2D mass spectrum. If the radius modulation is small compared to the fragmentation zone (black lines in Fig. 3d), then there is very little modulation of the ion abundance (precursor and fragment), and the intensity of the peaks in the spectrum after Fourier transformation (FT) according to *t*_1_ is very low. If the radius modulation is large compared to the fragmentation zone (grey line in Fig. 3d), then the evolution of the ion abundance shows periodic sharp peaks, and the peaks in the spectrum after FT according to *t*_1_ will have intense harmonics. If the radius modulation has the same range as the fragmentation zone (green line in Fig. 3d), then the evolution of the ion abundance is close to a sinusoid, and the peaks in the spectrum after FT according to *t*_1_ is at its maximum intensity and the harmonics are at low intensity (van Agthoven et al. 2014).

At the end of τ_m_, both precursor and fragment ions co-exist in the ICR cell at different radii. If the fragmentation method used during *τ*_m_ does not require pulsing a gas into the ICR cell (e.g. IRMPD or ECD), then the pressure in the ICR cell is sufficiently low that issues of coherence loss of the ion packet do not occur. The abundance of the fragment ions depends on the radius of the precursor ions at the end of P_2_. As a result, following Eq. 3, they are modulated according to *t*_1_ and $\omega_{\text{A}} = \omega_{\text{ICR}} - \omega_{\text{N}}^{\text{RF}}$, i.e. the cyclotron frequency of their precursors. When precursor ions are fragmented, their abundance decreases, which leads to a modulation of both precursor ion radii and precursor ion abundance according to *t*_1_ and according to $\omega_{\text{A}} = \omega_{\text{ICR}} - \omega_{\text{N}}^{\text{RF}}$ at the end of *τ*_m_. Precursor ion abundances follow a modulation which depends on Eq. 3, while fragment ion abundances follow $1 - r\left( {t_{1} } \right)$ (see Eq. 4).

After fragmentation, all ions in the ICR cell are excited by a third pulse P_3_ (see Fig. 3a, b), prior to detection. The frequency range of P_3_ needs to cover the cyclotron frequencies of all the fragment ions of interest. In one-dimensional FT-ICR MS, ions are excited from the centre of the ICR cell to the optimal radius for detection before detection. In 2D FT-ICR MS, ions are within a range of 2*r*_1_ at the start of P_3_. However, if 2*r*_1_ is significantly smaller than the radius of ions after P_3_, then the ions in the ICR cell at the end of *τ*_m_ can be assumed to be at the centre of the ICR cell and the effect of P_3_ can be assumed to be the same for all ions (van Agthoven et al. 2014).

In the 2D FT-ICR MS experiment, each iteration of the pulse sequence contains the following events:

- Ionization, external ion accumulation, and ion transfer to the ICR cell (0.05–1 s).
- P_1_-*t*_1_-P_2_ encoding sequence (typically a few ms).
- Fragmentation (10 ms–1 s).
- P_3_ excitation (20 ms).
- Transient acquisition (0.5 s).

Each iteration of the pulse sequence lasts between 0.6 and 2 s, approximately. The most important factors in the duration of an experiment are the number of scans, the ion accumulation, the fragmentation period, and the duration of the transient. With a continuous ion source such as electron impact ionization (EI), electrospray (ESI), nanospray (nanoESI), or atmospheric pressure photoionization (APPI), the amount of sample consumed is proportional to the duration of the experiment (van Agthoven et al. 2011b, 2015). For punctual ion sources such as matrix-assisted laser desorption ionization (MALDI), the amount of sample consumed only depends on the number of laser shots accumulated.

The acquisition of a dataset for a 2D FT-ICR MS experiment is set at regularly incremented values of *t*_1_. The number of data points and their time increment in each dimension determine the mass range for the precursors and the fragments, the total experimental time, as well as the resolving power and signal-to-noise ratio of each peak. For each value of *t*_1_, a transient is measured, acquired, and saved to obtain a 2D dataset. The dataset acquired from a 2D FT-ICR MS experiment can be expressed as $S\left( {t_{1} ,t_{2} } \right)$, where *t*_1_ is the duration of the delay in the sequence, during which the precursor ions have been let to evolve, and *t*_2_ corresponds to the transient detection period. Both *t*_1_ and *t*_2_ have similar roles in the expression of the signal; however, they correspond to very different actions. Each value of *t*_1_ corresponds to a measure of a transient, while *t*_2_ is the classical time during the measurement of the transient (i.e. *t*_1_ is a delay and *t*_2_ is the date at which each transient point was measured).

To process a dataset from a two-dimensional mass spectrometry experiment, the FT of each transient according to *t*_2_ is calculated (and can be expressed as $S\left( {t_{1} ,\omega_{2} } \right)$), followed by the FT of the signal at each frequency in the resulting spectra according to *t*_1_ (and can be expressed as $S\left( {\omega_{1} ,\omega_{2} } \right)$). The *ω*_1_ frequency measures the cyclotron frequency of the precursor ions and the *ω*_2_ frequency measures the cyclotron frequency of the fragment ions. Therefore, the *ω*_1_ dimension is called the precursor ion dimension and the *ω*_2_ dimension is called the fragment ion dimension (van Agthoven et al. 2013). One program that has been developed with 2D MS in mind is SPIKE (Spectroscopy Processing Innovative KErnel), an open source Python package dedicated to Fourier spectroscopies (Chiron et al. 2016).

To optimize the quality of the 2D spectrum, each transient, both in *t*_1_ and *t*_2_, is apodized and subsequently zerofilled. Zerofilling consists in extending the experimental transient with null values. Without zerofilling, the FT of an N real points dataset produces only N/2 useful information, and loses information. By at least doubling the size of the transient (i.e. “zerofilling once”), all spectral information is recovered. In 2D MS, zerofilling has to be performed at least once along each dimension, which means that the size of the dataset is temporarily multiplied by 4 during the processing (Marshall and Verdun 1990).

Apodization is performed to compensate for the fact that a dataset has a finite duration. The end of a dataset can be modelled by multiplying the dataset by a box function, which leads to convoluting the spectrum by a sin(*x*)/*x* (also called sinc(*x*)) function and therefore “wriggles” or “feet” distorting each peak in the spectrum. Apodization corresponds to the multiplication of each transient by a windowing function which smooths the ends of the transient and minimizes the wriggles on the sides of each peak. In 2D FT-ICR MS processing, apodization is performed on each transient before FT according to *t*_2_, and then on $S\left( {t_{1} ,\omega_{2} } \right)$ before FT according to *t*_1_ (Marshall and Verdun 1990). Many apodization windows exist currently in SPIKE. Apodization by a Kaiser (*β*) window seems to give the best results, with *β* = 3.5 along *t*_2_ and *β* = 5 along *t*_1_ (Kuo 1966).

Every data point in $S\left( {t_{1} ,t_{2} } \right)$ is real. After the first FT according to *t*_2_, the data points of the resulting dataset $S\left( {t_{1} ,\omega_{2} } \right)$ are complex. Because the evolution in *t*_1_ comes from a different phenomenon than the modulation in *t*_2_, the amplitude is modulated (see Eq 4). In consequence, the real and imaginary components of $S\left( {t_{1} ,\omega_{2} } \right)$ have to be transformed independently as two independent real series. After this second FT according to *t*_1_, the data points of the resulting dataset $S\left( {\omega_{1} ,\omega_{2} } \right)$ have four components: ${\text{RR}}\left( {\omega_{1} ,\omega_{2} } \right)$, ${\text{RI}}\left( {\omega_{1} ,\omega_{2} } \right)$, ${\text{IR}}\left( {\omega_{1} ,\omega_{2} } \right)$, and ${\text{II}}\left( {\omega_{1} ,\omega_{2} } \right). S\left( {\omega_{1} ,\omega_{2} } \right)$ is expressed as a hypercomplex number (Delsuc 1988; Ernst et al. 1987):

5 $$S\left( {\omega_{1} ,\omega_{2} } \right) = {\text{RR}}\left( {\omega_{1} ,\omega_{2} } \right) + i{\text{RI}}\left( {\omega_{1} ,\omega_{2} } \right) + j{\text{IR}}\left( {\omega_{1} ,\omega_{2} } \right) + k{\text{II}}\left( {\omega_{1} ,\omega_{2} } \right),$$

With

6 $$i^{2} = j^{2} = - 1$$

7 $$k^{2} = 1$$

The hypercomplex values *i*, *j*, and *k* follow these rules:

8 $$i \cdot j = j \cdot i = k$$

9 $$i \cdot k = k \cdot i = - j$$

10 $$j \cdot k = k \cdot j = - i$$

The magnitude mode spectrum $M\left( {\omega_{1} ,\omega_{2} } \right)$ can be obtained using the hypercomplex modulus for each data point:

11 $$M\left( {\omega_{1} ,\omega_{2} } \right) = \sqrt {{\text{RR}}\left( {\omega_{1} ,\omega_{2} } \right)^{2} + {\text{RI}}\left( {\omega_{1} ,\omega_{2} } \right)^{2} + {\text{IR}}\left( {\omega_{1} ,\omega_{2} } \right)^{2} + {\text{II}}\left( {\omega_{1} ,\omega_{2} } \right)^{2} }$$

The Nyquist frequencies, which correspond to the highest frequencies detected, are determined by the following equations:

12 $$f_{N1} = \frac{1}{{2\Delta t_{1} }}$$

and

13 $$f_{N2} = \frac{1}{{2\Delta t_{2} }}$$

in which *f*_*N*1_ is the Nyquist frequency in the precursor ion dimension, *Δt*_1_ the increment in *t*_1_, *f*_*N*2_ the Nyquist frequency in the fragment ion dimension, and *Δt*_2_ the increment in the acquisition of the transient *t*_2_. The *f*_*N*1_ frequency determines the lowest precursor *m*/*z* ratio and the *f*_*N*2_ frequency the lowest fragment *m*/*z* ratio.

The lowest frequency in excitation pulses is of the order of several 10 kHz. For example, on an FT-ICR mass spectrometer with a 12 T magnet, an excitation pulse for a mass range with a maximum *m*/*z* ratio of *m*/*z* 3000, the lowest frequency is 56.4 kHz. Any ion with a cyclotron frequency below this value does not get excited. In the 2D FT-ICR MS pulse sequence, the radius of such an ion does not get modulated. As a result, the lowest useful frequency in a 2D mass spectrum is determined by the lowest frequency in the excitation pulse (P_1_ and P_2_ for precursor ions, and P_3_ for fragment ions).

An additional correction step is required during the processing of the vertical axis because of the frequency correction − *ω*_N_^RF^ term which appears in Eq. 3 as a correction of the continuous phase pulse generator. This additional frequency produces a phase modulation of the precursor signal along *t*_1_ superimposed to the amplitude modulation produced by the fragmentation event and described by Eq. 4. Uncorrected, this modulation splits all frequencies by an offset ± *ω*_off_ with $\omega_{\text{off}} = \omega_{\text{N}}^{\text{RF}}$ in the precursor ion dimension.

For this reason, a digital demodulation of each signal as a function of *t*_1_ is performed in the data processing by multiplying each column in the dataset by $e^{{i\left( {\frac{\pi }{2}\omega_{\text{off}} t_{1} - \frac{\pi }{2}} \right)}}$ before the FT according to *t*_1_. The removal of this frequency splitting allows a √2 gain in signal-to-noise ratio. As a further consequence, unmodulated signals (such as non-fragmenting ions) appear at the *ω*_off_ frequency rather than at the zero frequency, and appear in the final spectrum as a strong horizontal line, located at *ω*_off_, i.e. at the highest excited mass. In the same manner, the highest (Nyquist) measured frequency is lowered by the same amount. Frequency-to-mass conversion following Eq. 2 can be applied to both dimensions of the 2D mass spectrum.

The resolving power at *m*/*z* 400 in a 2D mass spectrum depends on the intrinsic resolution of the instrument (i.e. the magnetic field), the experimental resolution (i.e. mostly the pressure in the ICR cell), and the measurement (i.e. the duration of the acquisition and the increment in delay or measurement time). The resolving power in a 2D mass spectrum can be separated into two values: the vertical precursor ion resolving power and the horizontal fragment ion resolving power. In both dimensions, the resolving power increases with the magnetic field and decreases when the pressure in the ICR cell increases. Because in both dimensions the spectrum is obtained using an FT, the resolving power is proportional to the frequency and inversely proportional to the *m*/*z* ratio. The resolving power in each dimension is proportional to the maximum values of *t*_1_ and *t*_2_ (i.e. total acquisition time). In the fragment ion dimension, the resolving power indicates the capacity to separate the *m*/*z* ratios of fragment ions. In the precursor ion dimension, the resolving power indicates the accuracy of the correlation between precursor and fragment ions (van Agthoven et al. 2016).

At a given resolving power, the number of data points can be decreased by increasing the low mass limit (i.e. decreasing the Nyquist frequency). For a given acquisition time and number of data points, a choice can be made to favour the resolving power in one dimension or the other. For example, in bottom-up proteomics, the mass spectrum of the precursor ions is very dense and separation in the vertical precursor ion dimension is crucial. However, the fragment ion scans are typically less dense. A large number of data points in the vertical precursor ion dimension (e.g. 4096) is therefore more important than a large number of data points in the horizontal fragment ion dimension (e.g. 262,144). In top-down proteomics, the complexity of the fragment ion scans demands a large number of data points in the horizontal fragment ion dimension (e.g. 4,194,304) (Floris et al. 2016). Finally, there is one degree of freedom possible with non-uniform sampling, where skipping data points along *t*_1_ can significantly enhance the resolving power in the vertical precursor dimension without increasing the total acquisition time (Bray et al. 2017).

The resolving power can also be seen as the maximum number of independent signals that can be resolved in a spectrum. The maximum number of signals resolvable in 2D MS is the product of both resolving powers of precursor and fragment axes. This third value that we can call 2D resolving power is a large number, usually well over 10^7^.

### Interpretation of two-dimensional mass spectra

**Fig. 4:**
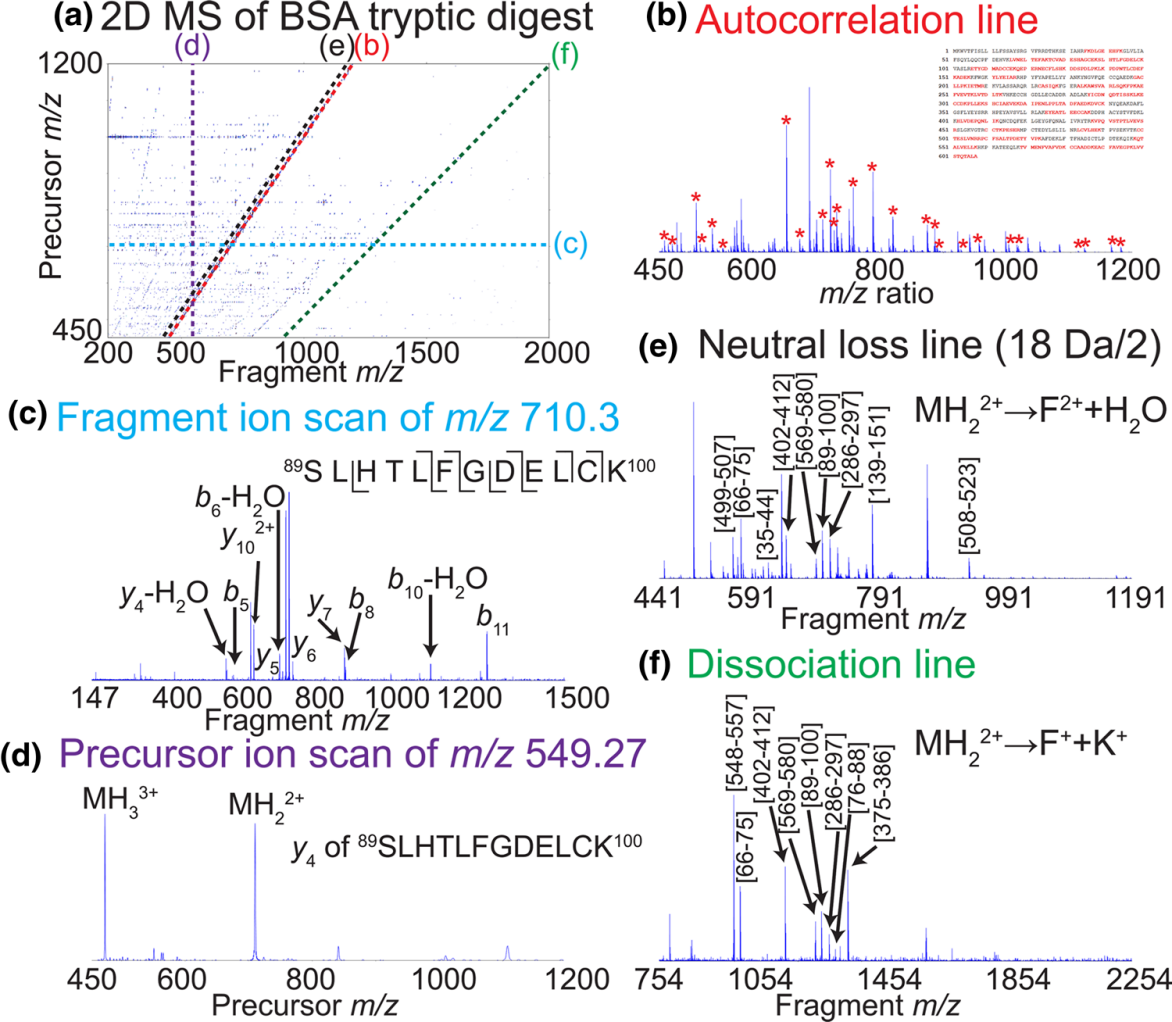
**a** 2D IRMPD mass spectrum of bovine serum albumin tryptic digest. Each peak corresponds to a dissociation, with the *m/z* ratio of the precursor plotted vertically and the *m*/*z* ratio of the fragment plotted horizontally. **b** Autocorrelation line with assigned peaks and sequence coverage. The autocorrelation line shows the *m*/*z* ratios of the precursor ions. **c** Fragment ion scan of *m/z* 710.3. The fragment ion scan of each precursor ion can be extracted horizontally. **d** Precursor ion scan of *m/z* 549.27. The precursor ion scan of each fragment ion can be extracted vertically. **e** Neutral loss line of water by doubly charged precursors. Each neutral loss line can be extracted as a line that is parallel to the autocorrelation line. **f** Dissociation line for loss of lysine with one charge loss for doubly charged precursors. Each dissociation line is characterized by the charge states of the precursor and the fragment ions and by the mass that is lost

After data processing, the 2D mass spectrum can be viewed in the *m*/*z* ratio domain. Figure 4 shows the 2D mass spectrum of a tryptic digest of bovine serum albumin (BSA) with IRMPD fragmentation. Fragment ion *m*/*z* ratios can be read on the horizontal axis and precursor ion *m*/*z* ratios can be read on the vertical axis. Each peak corresponds to a fragmentation (van Agthoven et al. 2013).

The 2D mass spectrum (Fig. 4a) contains several characteristic lines. The autocorrelation line (extracted in Fig. 4b) corresponds to the modulation of the intensity of the precursor ions according to their own cyclotron frequency. The equation of the autocorrelation line is:

14 $$\left( {m/z} \right)_{p} = \left( {m/z} \right)_{f}$$

in which (*m*/*z*)_p_ is the precursor axis *m*/*z* ratio and (*m*/*z*)_f_ is the fragment axis *m*/*z* ratio. The autocorrelation line is not the mass spectrum of the precursor ions, because the intensity of the peaks depends on the modulation of the fragmentation efficiency during the ion radius modulation. If a precursor ion does not fragment, then it is not detected on the autocorrelation line, regardless of its abundance in the mass spectrum. The autocorrelation line of the 2D mass spectrum of the tryptic digest of BSA shows the tryptic peptides that have been obtained after digestion of the protein. With the autocorrelation line alone, a sequence coverage of 55% is obtained after protein database interrogation.

Each horizontal line in a 2D mass spectrum corresponds to the fragment ion scan of the precursor ion whose *m*/*z* ratio can be read on the vertical axis. Figure 4c shows the fragment ion scan extracted at *m*/*z* 710.3 and shows the fragmentation pattern of the doubly charged ion of peptide ^89^SLHTLFGDELCK^100^, which enables its sequencing.

Each vertical line in a 2D mass spectrum corresponds to the precursor ion scan of the fragment ion whose *m*/*z* ratio can be read on the horizontal axis. Figure 4d shows the precursor ion scan of *m*/*z* 549.27, which corresponds to the *y*_4_ fragment of peptide SLHTLFGDELCK. As can be seen on the precursor ion scan, this fragment has two precursors: the doubly charged ion and triply charged ion of peptide SLHTLFGDELCK.

Since each fragmentation can be characterized by a loss of mass and a loss of charge, it can be extracted in a line with the following equation (van Agthoven et al. 2016):

15 $$\left( {m/z} \right)_{p} = \frac{n - p}{n}\left( {m/z} \right)_{f} + \frac{M}{n}$$

in which (*m*/*z*)_p_ is the precursor axis *m*/*z* ratio, (*m*/*z*)_f_ is the fragment axis *m*/*z* ratio, *n* is the charge of the precursor ion, *p* is the charge loss during the fragmentation, and *M* is the mass loss during the fragmentation.

Equation 15 can be used for neutral losses as well (van Agthoven et al. 2015):

16 $$\left( {m/z} \right)_{p} = \left( {m/z} \right)_{f} + \frac{M}{n}$$

In the 2D mass spectrum, neutral loss lines are parallel to the autocorrelation line, and the distance between them measures the neutral loss mass and the charge of the precursors and fragments. Figure 4e shows the neutral loss line for loss of 18 Da for doubly charged ions (i.e. water loss). Water loss in IRMPD can be indicative of a glutamate residue in a peptide, and all the peptide ions assigned on the neutral loss line contain a glutamate residue.

Figure 4f shows the dissociation line for loss of lysine by doubly charged precursor ions. Because lysine is a charge-carrying residue, loss of lysine happens together with charge loss. This dissociation line shows the peptides that have a lysine at the C-terminus. Because dissociation lines cross each other, a useful test to determine if a peak on a dissociation line actually corresponds to the dissociation that is sought is to check the charge state of its isotopic distribution against the charge state of the dissociation line.

Equation 15 can also be adapted for electron capture lines in ECD, in which *p* electrons are captured and there is no mass change (the mass of an electron is considered negligible compared to the mass of the ions) (Floris et al. 2018c; van Agthoven et al. 2012):

17 $$\left( {m/z} \right)_{p} = \frac{n - p}{n}\left( {m/z} \right)_{f}$$

During fragmentation, isotopes from the precursor ion can end up in one fragment or its complementary fragment. For example, in the top-down analysis of ubiquitin in 2D IRMPD MS, the precursor ion MH_7_^7+^ can dissociate into *y*_18_^2+^ and its complementary fragment *b*_59_^5+^. The elemental composition is C_378_H_636_N_105_O_118_S for MH_7_^7+^, C_93_H_157_N_29_O_26_ for *y*_18_^2+^, and C_285_H_475_N_76_O_93_S for *b*_59_^5+^. Each ^13^C isotope of MH_7_^7+^ can be in *y*_18_^2+^ or *b*_59_^5+^ after fragmentation. The probability $p(y_{18}^{2 + } /{\text{MH}}_{7}^{7 + } )$ for a precursor isotopologue of MH_7_^7+^ containing a number *n* of ^13^C isotopes (with probability $p\left( {{\text{MH}}_{7}^{7 + } } \right)$ of containing *n*^13^C isotopes) to dissociate into fragment isotopologue of *y*_18_^2+^ containing *p *< *n*^13^C isotopes (with probability $p\left( {y_{18}^{2 + } } \right)$ corresponding to the probability of the fragment *y*_18_^2+^ containing the *p*^13^C isotopes) and complementary fragment isotopologue of *b*_59_^5+^ (with probability $p\left( {b_{59}^{5 + } } \right)$ corresponding to the probability of the fragment *b*_59_^5+^ containing the *n*-*p*^13^C isotopes) is:

18 $$p\left( {y_{18}^{2 + } /{\text{MH}}_{7}^{7 + } } \right) = \frac{{p\left( {y_{18}^{2 + } } \right) \times p\left( {b_{59}^{5 + } } \right)}}{{p\left( {{\text{MH}}_{7}^{7 + } } \right)}}$$

**Fig. 5:**
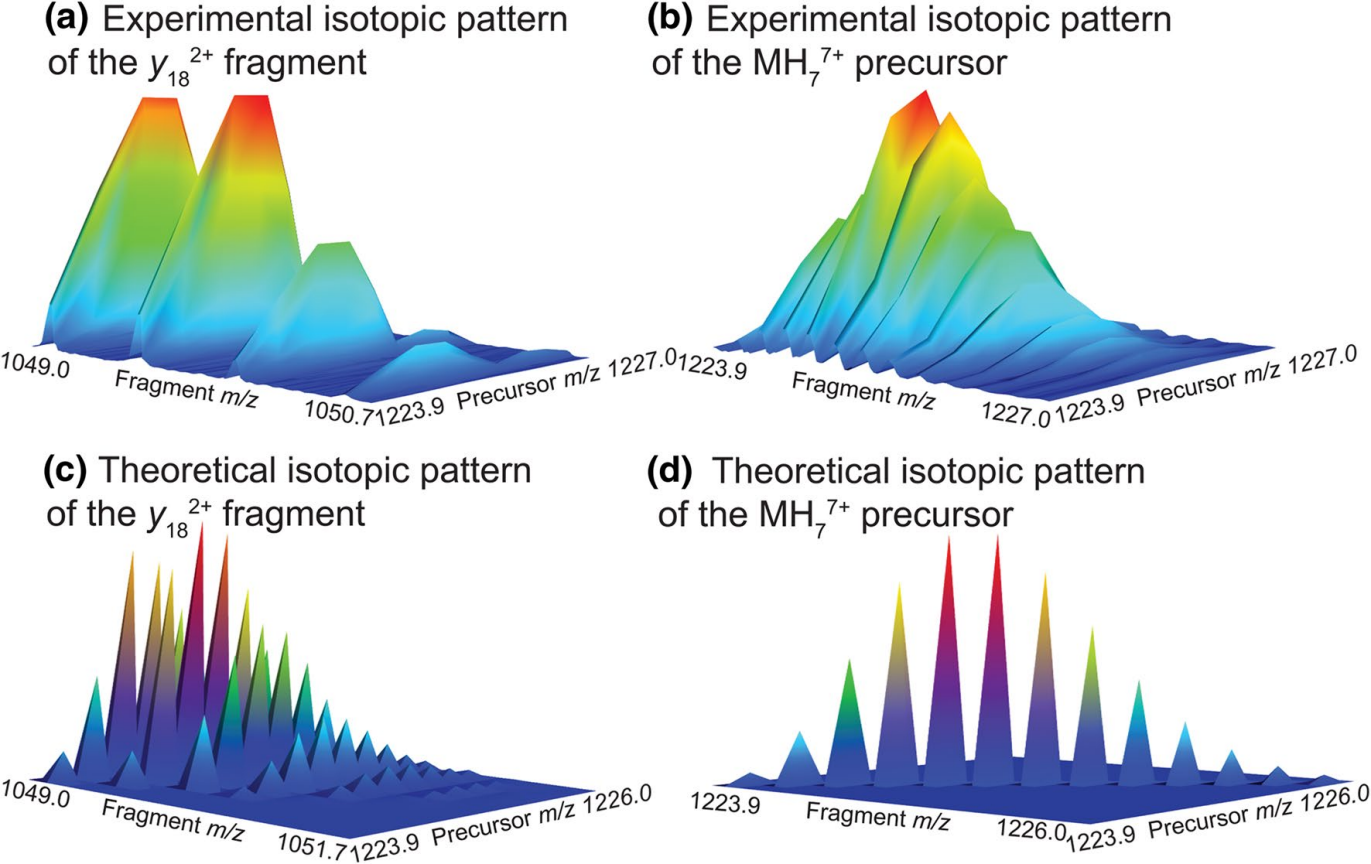
Three-dimensional representation of isotopic patterns in the 2D IRMPD mass spectrum of ubiquitin. **a** The experimental isotopic pattern of the *y*_18_^2+^ fragment ion (*m/z* 1049.10 for the monoisotopic peak) shows the that the contributions from the precursor isotopes in each peak in the isotopic pattern of the fragment ion cannot be separated due to the insufficient resolving power in the vertical dimension. **b** The experimental isotopic pattern of the MH_7_^7+^ precursor ion (*m/z* 1223.809664 for the monoisotopic peak) shows that the all the isotopic peaks of the precursor are on the autocorrelation line. **c** The theoretical isotopic pattern of the *y*_18_^2+^ fragment ion shows the contribution of each precursor isotope to the isotopic peaks of the fragment ion. **d** The theoretical isotopic pattern of the MH_7_^7+^ precursor ion shows the isotopic peaks of the precursor ion on the autocorrelation line

Equation 18 yields the theoretical isotopic distribution of the *y*_18_^2+^ fragment in the 2D mass spectrum. Figure 5 shows the three-dimensional representation of isotopic distributions from the 2D IRMPD mass spectrum of ubiquitin. Figure 5a shows the isotopic distribution of *y*_18_^2+^ from MH_7_^7+^, and Fig. 5b shows the isotopic distribution of MH_7_^7+^ on the autocorrelation line. Figure 5c shows the theoretical isotopic distribution for ^13^C isotopes for *y*_18_^2+^ from MH_7_^7+^, and Fig. 5d the theoretical isotopic distribution for ^13^C isotopes for MH_7_^7+^ on the autocorrelation line. Because the resolving power of the experimental 2D mass spectrum is limited along the precursor axis, the individual isotopic peaks cannot be separated in the experimental data, which explains the difference between the experimental and the theoretical distribution. As both the experimental and the theoretical distributions show, the spread of the isotopic distributions during a fragmentation is more complex than a simple dissociation line.

Neutral loss lines and dissociation lines can only be measured accurately along constant isotope lines (with the same number of ^13^C, ^15^N, ^17^O, ^18^O, ^2^H, and ^34^S in both the precursor and the fragment ion). However, while mapping isotopic distributions in a 2D mass spectrum requires taking Eq. 18 into account for the fragmentation of large molecules (e.g. proteins), isotopic distributions of peptides with less than 15 residues and small molecules can be considered as restricted to the dissociation line (O’Connor et al. 1996; O’Connor and McLafferty 1995).

**Fig. 6:**
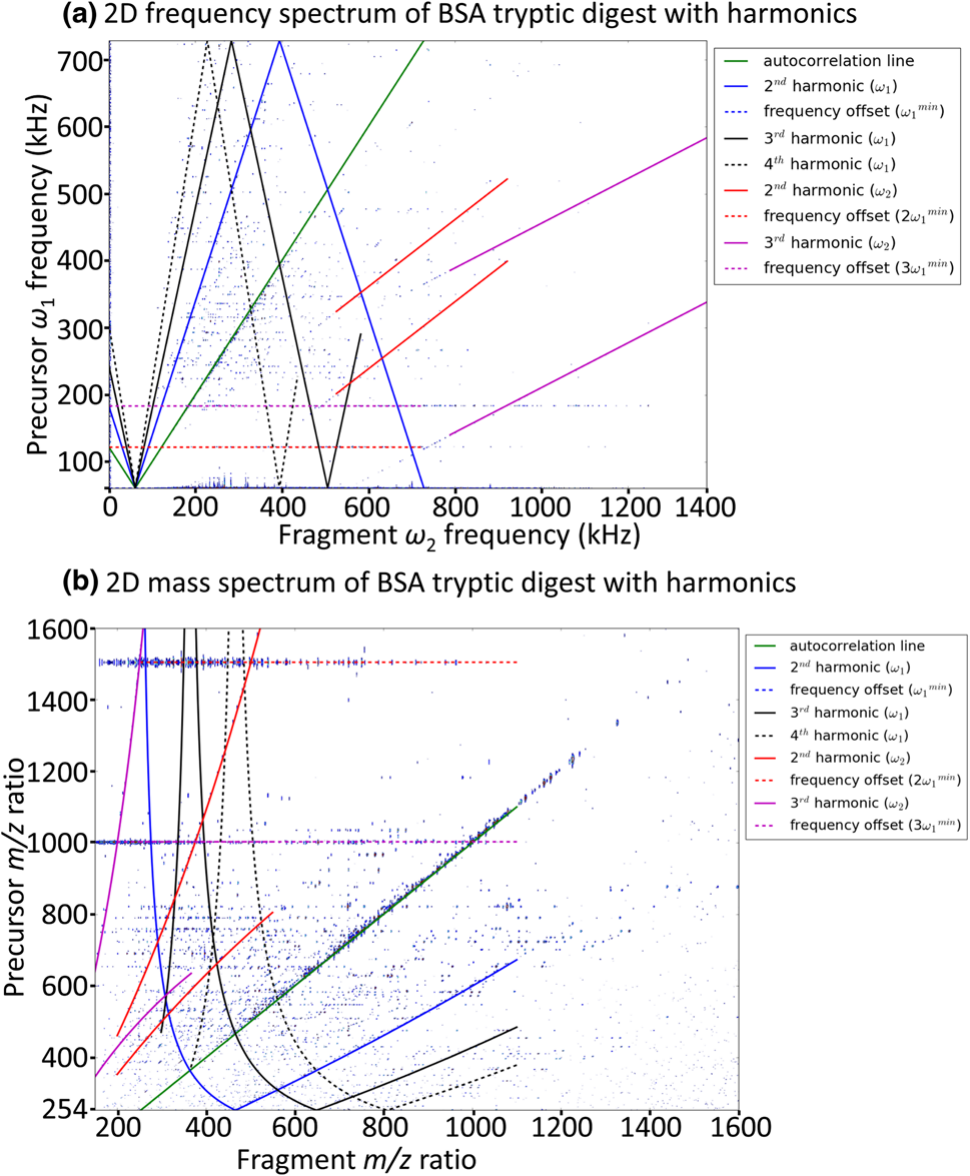
**a** 2D IRMPD spectrum of bovine serum albumin tryptic digest in the frequency domain. The position of the harmonics of the autocorrelation line are shown in both *ω*_1_ and *ω*_2_ axes, the rebound of the lines on the top and bottom of the frequency spectrum corresponds to the aliasing of the signal at frequencies higher than the Nyquist frequency. **b** 2D IRMPD spectrum in the *m/z* domain. The autocorrelation line is conserved but the other harmonics are curved, due to the inverse relationship between cyclotron frequencies and *m/z* ratio (cf. Eq. 1)

In any technique based on FT, any signal that is not perfectly sinusoidal generates harmonics at multiples of the ground frequency (or first harmonic) of the signal. In 2D MS, harmonics of the signal can occur in both the precursor and the fragment ion dimension. Figure 6 shows the 2D IRMPD spectrum of BSA from Fig. 4a in the frequency domain to show the harmonics in 2D MS.

In 2D mass spectra, harmonics are most visible as duplicates of the autocorrelation line as it is the location of the most intense signals. In frequency space, the equation of the autocorrelation line is:

19 $$\omega_{\text{v}} = \omega_{\text{h}} - \omega_{\text{off}}$$

in which *ω*_v_ is the modulation frequency of the precursor ion (plotted vertically), *ω*_h_ is its cyclotron frequency (plotted horizontally), and the offset *ω*_off_ is due to the frequency shift induced by the continuous phase pulse generator, corresponding to the lowest excited frequency in P_1_ (van Agthoven et al. 2014). In Fig. 6a, the autocorrelation line is shown to have a frequency offset of 61.4 kHz, which corresponds to *m*/*z* 3000 in the 12 T FT-ICR MS, which is the largest *m*/*z* ratio excited in the pulse sequence.

In the fragment dimension, harmonics are generated because of the non-sinusoidal nature of the mirror current on the detection plates. The shape of the ICR cell is optimized to limit the intensity of harmonics. In Fig. 6a, the horizontal harmonics of the autocorrelation line can be seen, with slopes of 1/2 for the second harmonic and 1/3 for the third harmonic, where the autocorrelation line itself is the first harmonic.

In the precursor dimension, harmonics of the autocorrelation line can be expressed as:

20 $$\omega_{\text{v}} = n\left( {\omega_{\text{h}} - \omega_{\text{off}} } \right)$$

in which *n* is an integer. In Fig. 6a, two vertical harmonics of the autocorrelation line can be seen, with *n* equalling 2 and 3.

In addition to harmonics, 2D mass spectra also feature folded over signals. Because their frequency *ω*_v_ is higher than the Nyquist frequency *ω*_N_, these signals appear at 2*ω*_N_ − *ω*_v_ instead of *ω*_v_. In frequency space, folded over harmonics of the autocorrelation line show up as lines with negative slopes (Marshall and Verdun 1990). Both the vertical harmonics discussed above fold over in the 2D spectrum.

In the conversion from frequency space to *m*/*z* ratio space, the only straight line that is conserved as a straight line is the identity line (i.e. the first harmonic of the autocorrelation line). Digital demodulation reduces the number of harmonics visible in the 2D mass spectrum, which improves the ease of interpretation of the 2D mass spectra (van Agthoven et al. 2012). In the *m*/*z* ratio domain, as shown in Fig. 6b, harmonics of the autocorrelation line can be easily recognized because they are not straight lines as the *m*/*z* ratios are a quasi-linear function of the inverse of frequencies in both dimensions. One side effect of digital demodulation is that they cause horizontal streaks at different multiples of *ω*_off_, which are the harmonics of the zero frequency now modulated by *ω*_off_. They can be seen in Fig. 6 at 61.4 kHz and 122.8 kHz.

The effect of digital demodulation is to subtract *ω*_off_ from the frequencies of each signal. As stated above, the continuous wave frequency synthesizer used for pulse generation adds a constant frequency *ω*_off_ to all signals along the vertical *ω*_1_ axis. A signal at a frequency *ω*_a_ + *ω*_off_ before digital demodulation will therefore have a frequency at *ω*_a_ after demodulation. Its second harmonic, which is at a frequency of 2(*ω*_a_ + *ω*_off_) before digital demodulation, will have a frequency of 2*ω*_a_ + *ω*_off_ after digital demodulation. While unavoidable, the presence of harmonics in a 2D mass spectrum can needlessly complicate data interpretation; harmonics have proven to be useful when using IR-ECD as a fragmentation method, by enabling the differentiation of N-terminal and C-terminal fragments for peptides (van Agthoven et al. 2018).

**Fig. 7:**
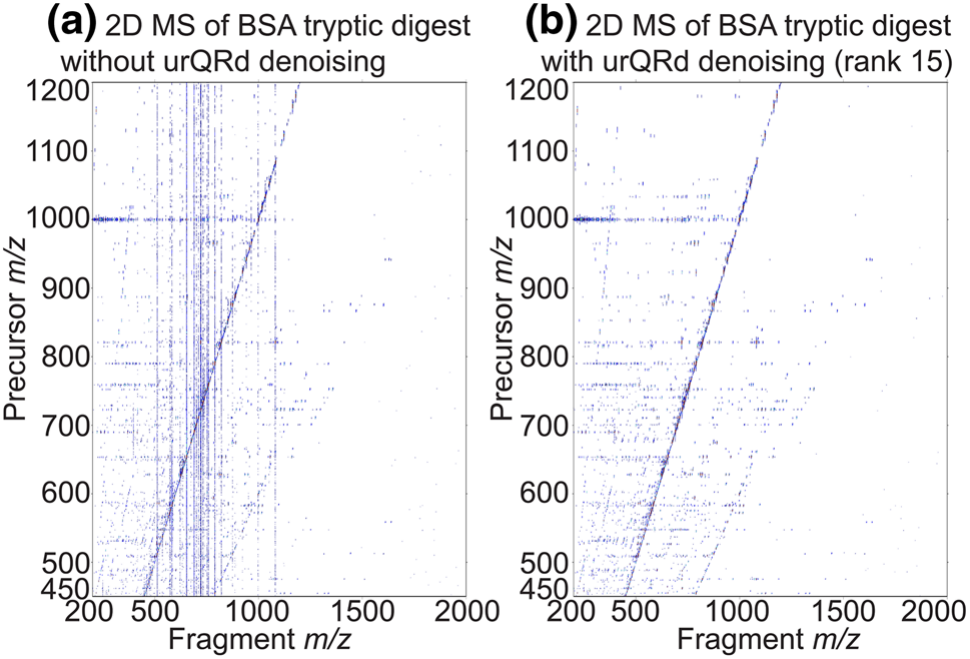
**a** 2D IRMPD mass spectrum of bovine serum albumin without denoising. **b** 2D IRMPD mass spectrum of bovine serum albumin after urQRd denoising (rank 15). The urQRd denoising removes the vertical noise stripes

2D mass spectra show vertical streaks along the peaks with the highest intensities. These streaks are called scintillation noise and are probably caused by fluctuations of the number of ions in the ICR cell (van der Rest and Marshall 2001). Figure 7a shows the 2D IRMPD mass spectrum of BSA shown in Fig. 4 with the scintillation noise. Scintillation noise is non-additive and roughly proportional to the signal *S*(t):

21 $$S\left( t \right) = A\left( {1 + n\left( t \right)} \right)\sqrt {1 + { \cos }\omega t}$$

in which *A* is the amplitude of the signal, *ω* its frequency, and *n*(*t*) the scintillation noise. Because of its proportionality to the signal, the impact of scintillation noise cannot be decreased by signal accumulation. Scintillation noise is an important cause of misinterpretation of 2D mass spectra (van Agthoven et al. 2011a).

Denoising algorithms can be applied to vertical columns before FT in the vertical dimension. The first denoising algorithm used for processing 2D MS is an algorithm initially proposed by Cadzow et al. and relies on data predictability, i.e. the fact that for each transient, in the absence of noise, there is a number *N* of data points, related to the number of signal present in the transient, after which it is possible to predict the (*N *+ 1)th data points (Cadzow and Wu 1987):

22 $$S_{N + 1} + \mathop \sum \limits_{k = 1}^{N} a_{k} S_{k} = 0$$

in which *a*_k_ are factors. An *n *× *p* sized Toeplitz matrix *T* can therefore be composed from the data points:

23 $$T = \left[ {\begin{array}{*{20}c} {\begin{array}{*{20}c} {S_{n + 1} } & {S_{n} } \\ {S_{n + 2} } & {S_{n + 1} } \\ \end{array} } & {\begin{array}{*{20}c} \cdots & {S_{1} } \\ \cdots & {S_{2} } \\ \end{array} } \\ {\begin{array}{*{20}c} \vdots & \vdots \\ {S_{n + p} } & {S_{n + p - 1} } \\ \end{array} } & {\begin{array}{*{20}c} \vdots & \vdots \\ \cdots & {S_{p} } \\ \end{array} } \\ \end{array} } \right]$$

*T* can be decomposed into eigenvalues and eigenvectors. If *n* is higher than *N*, then some eigenvalues of *T* will be zero. In the presence of noise, *T* has no eigenvalues that are equal to zero. The Cadzow algorithm sets the smallest eigenvalues of *T* to zero and reconstructs the denoised dataset from the denoised Toeplitz matrix.

The main advantage of the Cadzow algorithm is that no assumptions are made on the noise or signal power. The only parameter is the number of frequencies in the signal related to *N*/2, since this number determines how many eigenvalues correspond to the signal as opposed to noise. Unfortunately, in 2D MS, the number of frequencies is not known a priori and varies from column to column. Furthermore, the algorithm is very time-consuming and implies an SVD computation, with a total processing time nearly proportional to *n*^3^, in which *n* is the number of data points (van Agthoven et al. 2011a).

An alternative algorithm, called uncoiled random QR denoising (urQRd), relies on QR decomposition of a matrix randomly sampled from the data rather than an SVD decomposition. In the urQRd algorithm, the number of frequencies in the signal is not as crucial as in the Cadzow algorithm, making it a much more flexible than the Cadzow algorithm. The processing time for urQRd denoising is observed to be proportional to *n*^1.10^, which corresponds to a gain of almost three orders of magnitude in real cases. The urQRd denoising algorithm can therefore be applied to datasets of almost unlimited size (Chiron et al. 2014). Figure 7b shows the 2D IRMPD mass spectrum of BSA from Fig. 4 after denoising with the urQRd algorithm. A side-by-side comparison with the raw spectrum shows that urQRd is effective in removing noise signals without removing the useful signal.

## Conclusion

Two-dimensional mass spectrometry was first introduced in 1987, but it has only seen significant development since 2010, because of the computational demands to store and process datasets and the impact of scintillation noise on the spectra. Since then, the development of 2D MS and the urQRd algorithm have enabled applications in the analysis of small molecules, proteomics (both top-down and bottom-up), and polymers (Floris et al. 2016, 2017, 2018c; Simon et al. 2016; van Agthoven et al. 2015; van Agthoven et al. 2016). 2D MS has also been applied for MS^3^ experiments on proteins without ion isolation (Floris et al. 2018a, b). Because of the multiplexing of the data inherent to Fourier analysis, 2D MS has capabilities that other DDA and DIA techniques do not have in terms of correlation between precursor and fragment ions, especially for overlapping isotopic distributions (van Agthoven et al. 2016).

As an analytical technique, 2D MS is still in development. Alternative pulse sequences using stored waveform ion radius modulation (SWIM) enable the development of 2D MS in mass analysers other than the FT-ICR MS (Ross et al. 1993, 2002; van Agthoven and O’Connor 2017). Techniques that approach 2D MS are being developed for quadrupolar ion traps (Snyder et al. 2018). Non-uniform sampling acquisition has been shown to increase resolving power and shorten experiment durations (Bray et al. 2017). Theoretical studies enable a better understanding of the phenomena at play in a two-dimensional mass spectrometry experiment (Sehgal et al. 2016). Two-dimensional mass spectrometry has the potential to open up the entire field of mass spectrometry and to increase the structural information available on complex samples by several orders of magnitude.
